# Astragaloside IV accelerates hematopoietic reconstruction by improving the AMPK/PGC1α-mediated mitochondrial function in hematopoietic stem cells

**DOI:** 10.1186/s13020-025-01092-3

**Published:** 2025-04-01

**Authors:** Ling Zhang, Wanqi Xu, Yueying Zeng, Long Wang, Jiesi Luo, Xiaogang Zhou, Qibing Mei, Dalian Qin, Anguo Wu, Jianming Wu, Feihong Huang

**Affiliations:** 1 Luzhou Key Laboratory of Activity Screening and Druggability Evaluation for Chinese Materia Medica, School of Pharmacy, Southwest Medical University, Luzhou, 646000 China; 2 School of Basic Medical Sciences, Southwest Medical University, Luzhou, 646000 China

**Keywords:** Astragaloside IV, Hematopoietic stem cells, Mitochondrial function, AMPK/PGC1α pathway

## Abstract

**Background:**

Radiotherapy can damage hematopoietic stem cells (HSC) in bone marrow, leading to impaired hematopoietic function. Current treatments mainly target differentiated hematopoietic progenitor cells, which may accelerate their depletion. Astragaloside IV (AS-IV), derived from Astragalus membranaceus, shows potential in hematopoiesis, but its direct effects on HSC remain unclear.

**Methods:**

The study employed both in vitro and in vivo approaches. In vitro experiments utilized K562 cells and mouse bone marrow nucleated cells (BMNCs) to evaluate AS-IV's effects on cell proliferation and mitochondrial function. In vivo studies involved a 4.0 Gy total body irradiation mouse model treated with different doses of AS-IV (50 mg/kg and 100 mg/kg). The mechanism of action was investigated through Western blot, flow cytometry, and metabolomics analyses. The AMPK/PGC1α pathway regulation was verified using AMPK inhibitors and mutant plasmid, with molecular docking confirming AS-IV’s direct binding to AMPK.

**Results:**

In vitro studies demonstrated that AS-IV significantly promoted the proliferation of K562 cells and BMNC while enhancing their mitochondrial membrane potential, mitochondrial mass, and ATP production. In the irradiated mouse model, AS-IV treatment led to significant improvements in peripheral blood cell counts, including white blood cells, red blood cells, and hemoglobin levels. Further investigation revealed that AS-IV increased the proportion of HSC in both bone marrow and spleen while improving their mitochondrial function. Transcriptomic sequencing and Western blot analysis identified the AMPK/PGC1α signaling pathway as the key mechanism underlying AS-IV-mediated mitochondrial enhancement. These findings were validated through pharmacological inhibition of AMPK and AMPK^K45R^ mutation experiments.

**Conclusion:**

AS-IV accelerates hematopoietic reconstruction following radiation injury via activation of the AMPK/PGC1α signaling pathway, which enhances HSC mitochondrial function.

**Supplementary Information:**

The online version contains supplementary material available at 10.1186/s13020-025-01092-3.

## Introduction

The rising incidence and mortality rates of cancer have made it the leading cause of death in our country, representing a significant public health challenge [1]. Radiotherapy, a widely used cancer treatment, effectively targets tumor cells but can also damage normal cells, particularly the rapidly dividing hematopoietic cells in the bone marrow [2–4]. This damage often results in impaired hematopoietic function, a common and serious complication that limits the dosage and duration of radiotherapy [5, 6]. Current clinical interventions for managing post-radiotherapy bone marrow damage, such as colony-stimulating factors, recombinant proteins, and blood transfusions, mainly target highly differentiated hematopoietic progenitor cells [7, 8]. Although these treatments offer rapid relief, prolonged use may deplete hematopoietic stem and progenitor cells or cause drug tolerance, reducing treatment efficacy [9, 10]. Consequently, there is an urgent need for therapies that synergistically stimulate transient progenitor expansion and preserve HSC self-renewal capacity.

Hematopoietic stem cells (HSC) are the progenitor cells responsible for the generation and replenishment of all blood cell types [11]. Under normal conditions, HSC predominantly resides in a quiescent state (G0 phase), maintaining a reserve with minimal mitochondrial activity and metabolic function [12]. However, in response to hematopoietic stress, these quiescent HSC become activated, entering the cell cycle with increased mitochondrial function and a shift towards oxidative phosphorylation (OXPHOS) as their primary energy source [13, 14]. HSC are characterized by a higher mitochondrial mass and membrane potential compared to more mature hematopoietic cells, attributes that are closely linked with enhanced stem cell function [15–17]. Moreover, mitochondrial membrane potential (MMP) serves as a valuable marker for identifying HSC with enhanced functional potency [18]. Lkb1 regulates HSC energy balance and growth through AMPK activation. Additionally, energy metabolites like α-ketoglutarate and citric acid enhance HSC differentiation by affecting histone and DNA methylation [19]. Importantly, studies have indicated that boosting mitochondrial function, such as through mitochondria-targeted coenzyme Q10 (Mito-Q), can enhance hematopoiesis, especially in aging HSC [20]. As a result, boosting mitochondrial function in HSC is a promising strategy to enhance hematopoietic recovery.

*Astragalus membranaceus*, a widely used traditional Chinese medicine, is known for its Qi tonifying properties and hematopoietic stimulating effects [21]. Its use dates back over two millennia in China, documented in Shennong's Classic of Materia Medica [22]. *Astragalus membranaceus* is recognized as a significant Qi-tonifying herb within the framework of Traditional Chinese Medicine (TCM). It is utilized in clinical settings for its capabilities in immunomodulation, stimulating hematopoiesis, anti-fatigue, restorative, and hypoglycemic activity [23–25]. In TCM, tonifying Qi therapies restore hematopoietic function by enhancing organ-tissue energy metabolism, a mechanism analogous to mitochondrial bioenergetic regulation [26]. However, no direct evidence exists regarding astragalus' modulation of mitochondrial dynamics in HSC, particularly under hematopoietic stress conditions.

Astragaloside IV (AS-IV), the principal active component of *Astragalus membranaceus*, exhibits anti-inflammatory, anti-diabetic, immune-regulatory, and cardioprotective effects via multiple signaling pathways [27, 28]. Reports indicate that AS-IV promotes hematopoiesis through the upregulation of hematopoietic growth factors within the bone marrow microenvironment [29]. Nevertheless, the direct impact of AS-IV on HSC remains inadequately explored. This study mechanistically deciphers the efficacy of AS-IV in enhancing mitochondrial function in HSC to promote hematopoietic recovery. Additionally, by integrating traditional Chinese medicine with contemporary stem cell research, this study provides translational evidence bridging TCM theory with modern stem cell biology.

## Materials and methods

### Chemicals

AS-IV (CAS: 83207-58-3, 98% purity by HPLC) was procured from Shanghai Jizi Biochemical Technology Co., Ltd. (Shanghai, China) and reconstituted following the manufacturer's guidelines.

### Cell culture

K562 cells, used as a model for chronic myeloid leukemia (CML), were obtained from the U.S. Type Culture Collection in Bethesda, Maryland, USA. They were grown in RPMI-1640 medium (Gibco, Invitrogen Corporation, Carlsbad, CA, USA) with the addition of 10% fetal bovine serum (Sperikon Life Sciences & Biotechnology Co., Ltd., Chengdu, China) and 1% penicillin–streptomycin (SPERIKON, Chengdu, China). The cells were cultured in an incubator maintained at 37 °C with 95% humidity and 5% CO₂.

### Cell counting kit-8 assay

Cell viability and proliferation were assessed using the CCK-8 assay (APExBIO Technology LLC, Shanghai, China). Cells (5 × 10^3^/well) were treated with AS-IV (1.25–40 μM, 24–48 h). Post-treatment, 20 μL CCK-8 reagent was added, incubated for 2 h, and absorbance measured at 450 nm (BioTek, IL, USA).

### Bone marrow nucleated cells extraction

Referred to the previous experimental method [30], femurs from SPF KuMing (KM) mice were flushed with RPMI-1640. Mononuclear cells were isolated via density gradient centrifugation (400 ×*g*, 30 min) using mononuclear cell isolation solution (Solarbio, Beijing, China).

### Mitochondrial analyses

*Mitochondrial membrane potential analysis (MMP)* K562 cells were exposed to AS-IV at 2.5, 5, and 10 μM for 5 days, while BMNC were treated under the same conditions for 9 days. Additionally, bone marrow (BM) and spleen (SP) cells were collected from mice after radiation injury and washed twice with phosphate-buffered saline (PBS). JC-1 staining (Beyotime Biotechnology, Shanghai, China) followed by flow cytometer (BD Biosciences, San Jose, CA, USA) or an inverted fluorescence microscope (Nikon, Tokyo, Japan) [31]. The red-to-green fluorescence ratio was compared across treatment groups.

*Mitochondrial content* MitoTracker Green (Beyotime Biotechnology, Shanghai, China) staining analyzed via fluorescence microscopy or flow cytometry.

*ATP measurement* ATP concentrations in K562 cells and the BM and SP of irradiated mice were measured using an ATP detection kit (Beyotime Biotechnology, Shanghai, China). The tissues were subjected to lysis using ATP lysis buffer, and the protein content was measured and homogenized in each sample with the lysis buffer. Subsequently, ATP levels were quantified employing a microplate reader (BioTek, Winooski, VT, USA).

### Quantitative real-time PCR (qRT-PCR)

Total RNA from AS-IV-treated K562 cells (10 μM) was extracted with TRIzol (Takara, Kusatsu, Japan). cDNA synthesis used 1 μg RNA with a One-Step cDNA Kit (Vazyme, Nanjing, China). qRT-PCR was performed using SYBR Green Master Mix (Vazyme, Nanjing, China) on a Bio-Rad CFX96 system. Data normalized to GAPDH (primers in Supplementary Table 1).

### Colony formation

Cells (1 × 10^5^/mL) in MethoCult™ GF M3434 semi-solid medium (1 mL; STEMCELL, Shanghai, China) were cultured. A blank control group was established alongside AS-IV treatment groups at concentrations of 2.5, 5, and 10 μM, with each group containing three replicates. Follow STEMCELL's instructions for Mouse Colony Forming Unit (CFU) Assays to observe and count under a microscope.

### Animal studies

Two-month-old KuMing (KM) mice (18–22 g) were obtained from Dashuo Biotechnology Co., Ltd. (Chengdu, Sichuan, China) under SPF conditions. Each experimental group comprised six male and six female mice, which were randomly allocated to one of the following categories: control group, model group, erythropoietin (EPO) group (2000 U/kg), and AS-IV treated groups (50 and 100 mg/kg). With the exception of the normal control group, all mice received a single dose of 4 Gy total body irradiation (TBI) [32]. Following irradiation, both the control and model groups were continuously administered pure water via gavage for 13 days. Conversely, the remaining groups received either EPO or various concentrations of AS-IV for 13 consecutive days. On day 0, 7, 10 and 13, 40 μL peripheral blood of the fundus venous plexus was taken and mixed with 160 μL diluent. Use a fully automated blood cell analyzer (XT-1800i, Sysmex Corporation, Kobe, Japan) for measurement.

### RNA sequencing and data analysis

RNA was extracted from bone marrow cells of the normal control, model, and high-dose AS-IV (100 mg/kg) groups using TRIzol reagent (Takara, Kusatsu, Japan). RNA samples were stored at − 20 °C and transported on dry ice to Mallobio Biohazard Technology Co., Ltd. (Shanghai, China) for transcriptomic sequencing. RNA quality was assessed using a 2100 Bioanalyzer (Agilent, California, USA) and quantified with a Nanodrop 2000. Libraries were prepared with Illumina's TruSeq RNA Sample Preparation Kit using 1 μg total RNA and sequenced on an Illumina HiSeq X Ten/NovaSeq 6000 following library quantification with TBS380. Functional enrichment analyses were conducted employing Gene Ontology (GO) methodologies to identify differentially expressed genes (DEGs) that are associated with fundamental biological functions as categorized in the GO database. GO terms and KEGG pathways demonstrating a p-value of less than 0.05 were deemed significantly enriched with regard to DEGs.

### Flow cytometry

BM or spleen cells stained with lineage-specific antibodies were analyzed on flow cytometry (BD Biosciences, San Jose, CA, USA) [33]. The specific antibodies utilized in the flow cytometry assays were as follows: FITC-conjugated anti-CD3 (E-AB-F1013C), CD11b (E-AB-F1081C), CD45R (E-AB-F1112C), Gr-1 (E-AB-F1120C), and Ter119 (E-AB-F1125C); PE-conjugated anti-Sca-1 (E-AB-F1191D); APC-conjugated anti-c-kit (E-AB-F1092E); PE-Cy7-conjugated anti-CD127 (E-AB-F1023E); PE-Cy5. 5-conjugated anti-CD16/32 (E-AB-F0997E); and APC-Cy7-conjugated anti-CD34 (E-AB-F1284E). All antibodies utilized in this study were obtained from Elabscience (Wuhan, China).

### Metabolites analysis

Metabolites from BM or spleen cells were extracted with acetonitrile, derivatized (3-NPH/EDC), and analyzed on Waters UPLC-AB SCIEX 5500 system with HSS T3 column (100 mm × 2.1 mm, 1.8 µm) at a flow rate of 0.3 mL/min and a temperature of 40 °C.

### Western blotting

Proteins extracted with RIPA buffer (CST, MA, USA) containing protease/phosphatase inhibitors were resolved by SDS-PAGE and transferred to PVDF membranes. After blocking, membranes were incubated with primary (4 °C, 16 h) and secondary antibodies (RT, 1 h). ECL-based detection (4A Biotechnology Co., Ltd., Beijing, China) was performed on ChemiDoc™ Touch (Bio-Rad, Berkeley, CA, USA), with band intensity quantified using ImageJ. The primary antibodies utilized in this investigation comprised p-AMPKα (7A3423S, Abmart, Shanghai, China), AMPKα (E-AB-30491, Elabscience, Wuhan, China), PGC1α (T56630S, Abmart, Shanghai, China), GAPDH (E-AB-40337, Elabscience, Wuhan, China), and β-actin (P30002M, Abmart, Shanghai, China).

### Molecular docking simulations

The three-dimensional structure of AS-IV was retrieved from the PubChem database, while the crystal structure of the AMPKα central target was obtained from the RCSB Protein Data Bank (PDB) [34]. AS-IV was docked to AMPKα using Surflex-Dock. The SFXC score, representing the total score, was utilized to assess the binding affinity between AS-IV and the AMPKα protein.

### CETSA/DARTS

*Cellular thermal shift assay (CETSA)* AS-IV-treated K562 cells were subjected to thermal gradient exposure (37–72 °C, 5 °C increments, 5 min), lysed, and centrifuged (12,000 × g, 15 min). AMPKα thermal stability was quantified via immunoblotting of soluble fractions.

*Drug affinity responsive target stability assay (DARTS)* Cell lysates pre-incubated with AS-IV or DMSO (1 h, 4 °C) underwent limited proteolysis using streptomyces proteinase (1:500–1:1500 dilution) at 40 °C for 10 min under serum-free conditions. A fivefold loading buffer was added to the sample and heated at 95 °C for another 10 min. Protease-resistant AMPKα complexes were analyzed by Western blot.

### Cell transfection

K562 cells (2 × 10⁶/well in 6-well plates) were transfected with target/control plasmids using Vazyme transfection reagent (Vazyme, Nanjing, China). Plasmid DNA (Addgene, Massachusetts, USA) was diluted in Opti-MEM, mixed with transfection reagent, and incubated to form complexes. Control cells received empty vector or non-targeting plasmid.

### Statistical analysis

All assays were conducted in triplicate to guarantee the unwavering quality and reproducibility of the results. Data comparisons were performed using Student's t-tests or one-way analysis of variance (ANOVA). A p-value of less than 0. 05 was considered indicative of statistical significance.

## Results

### AS-IV enhances mitochondrial function and proliferation in K562 cells in vitro

**Fig. 1:**
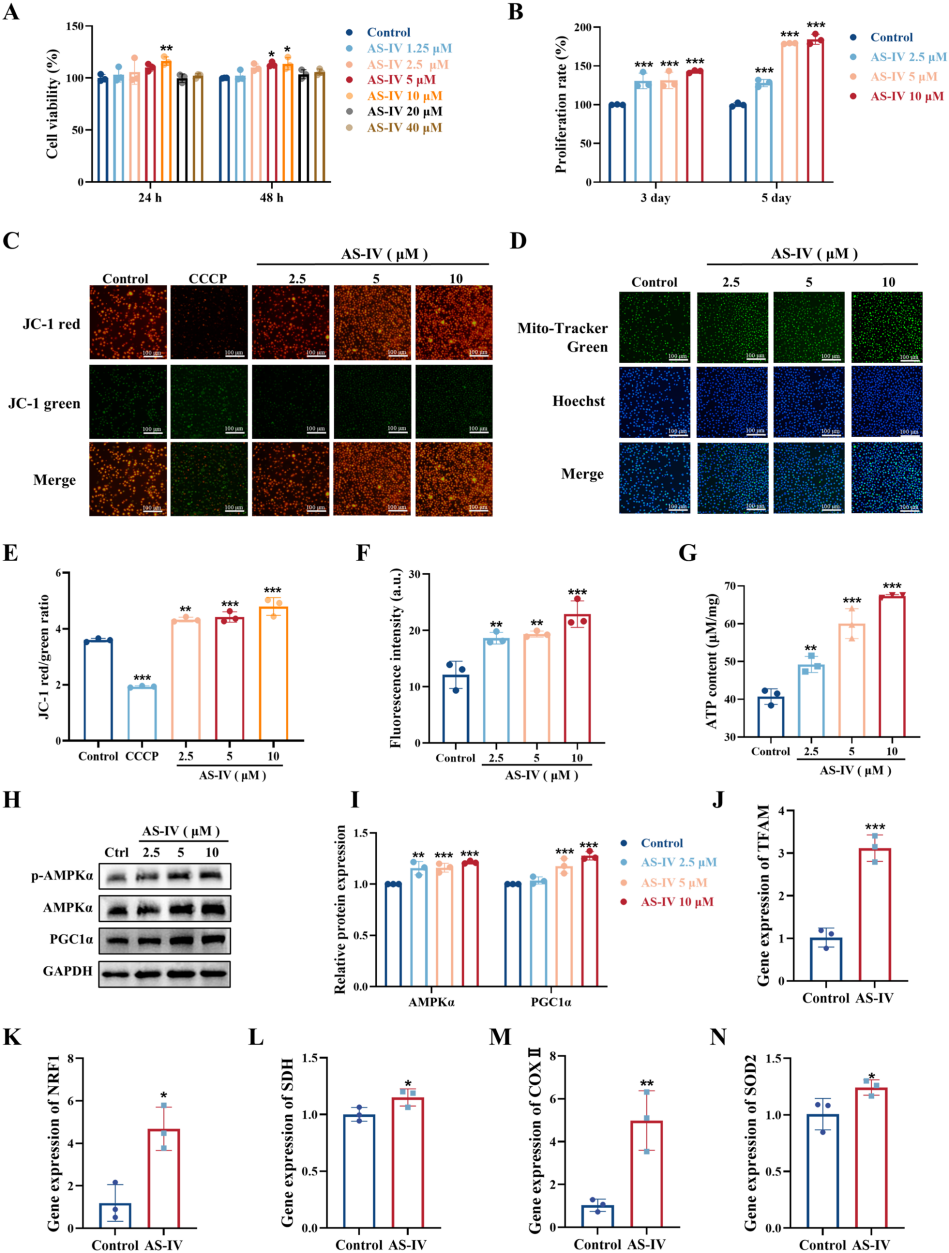
AS-IV enhances mitochondrial function and proliferation in K562 cells in vitro*.* **A** Effect of AS-IV on K562 cell viability. Effects of different time points and dosages on K562 cell viability (%); **B** Effect of AS-IV on K562 cells proliferation. Effect of different time points and dosages on the proliferation rate (%) of K562 cells; **C, E** K562 cells treated with the fluorescent probe JC-1 were labelled with AS-IV (2.5, 5, 10 μM) to assess mitochondrial membrane potential changes by inverted fluorescence microscopy. JC-1 polymer appears as red, whereas JC-1 monomer appears as green. Scale bar: 100 μm; **D**, **F** Mito-Tracker green fluorescent probe for detection of mitochondrial mass after drug administration. Nuclei were stained with Hoechst 33258 (Scale bar: 100 μm); **G** ATP Assay Kit detected the concentration of ATP produced by cells; **H**–**I** Representative immunoblot images and biochemical quantification of mitochondrial metabolism-associated pathway proteins (AMPK/PGC1α pathway) in K562 cells after 5 days treatment with AS-IV (2.5, 5, 10 μM). **J**–**N**. The mitochondrial function-related genes (TFAM, NRF1, SDH, COXII, SOD2) were verified by qRT-PCR. Data represent the mean ± standard deviation of three independent experiments. *p < 0.05, **p < 0.01, ***p < 0.001 compared with control

K562 cells, which represent primitive hematopoietic cells with the potential for multidirectional differentiation, serve as a model for investigating the effects of AS-IV on HSC [35]. We evaluated K562 cell activity following AS-IV treatment. The results of the CCK-8 assay indicated that AS-IV improved cell viability at concentrations of 5 μM and 10 μM in comparison to the control group without exhibiting cytotoxicity at concentrations below 40 μM (Fig. 1A–B). Microscopic observations further confirmed an increase in cell density with AS-IV treatment (Supplementary Fig. 1A), indicating that AS-IV promotes K562 cell proliferation.

In order to examine the impact of AS-IV on mitochondrial function, we assessed mitochondrial membrane potential, total mitochondrial mass, and ATP production in K562 cells. JC-1 staining demonstrated that AS-IV significantly increased red fluorescence intensity, indicative of an elevated mitochondrial membrane potential (Fig. 1C, E). Mito-Tracker Green assays showed an increase in mitochondrial mass (Fig. 1D, F), while ATP assays revealed elevated ATP levels following AS-IV treatment (Fig. 1G). Western blot analysis further indicated that AS-IV enhanced AMPKα phosphorylation and upregulated PGC1α expression, suggesting activation of the AMPK/PGC1α signaling pathway (Fig. 1H–I). To delineate AS-IV's effects on mitochondrial transcriptional regulation, we conducted qRT-PCR analysis of core bioenergetic markers. AS-IV treatment markedly upregulated mitochondrial biogenesis mediators (TFAM, NRF1) and oxidative phosphorylation components (SDH, COXII, SOD2), with expression levels quantified (Fig. 1J–N). Pharmacological challenge using CCCP-induced mitochondrial membrane depolarization in K562 cells. Notably, AS-IV treatment significantly ameliorated CCCP-induced mitochondrial damage in K562 cells and promoted recovery of ATP production (Supplementary Fig. 1B-D). These findings collectively suggested that AS-IV enhanced mitochondrial function and stimulated proliferation in K562 cells.

### AS-IV promotes mitochondrial function and proliferation of HSC in vitro

**Fig. 2:**
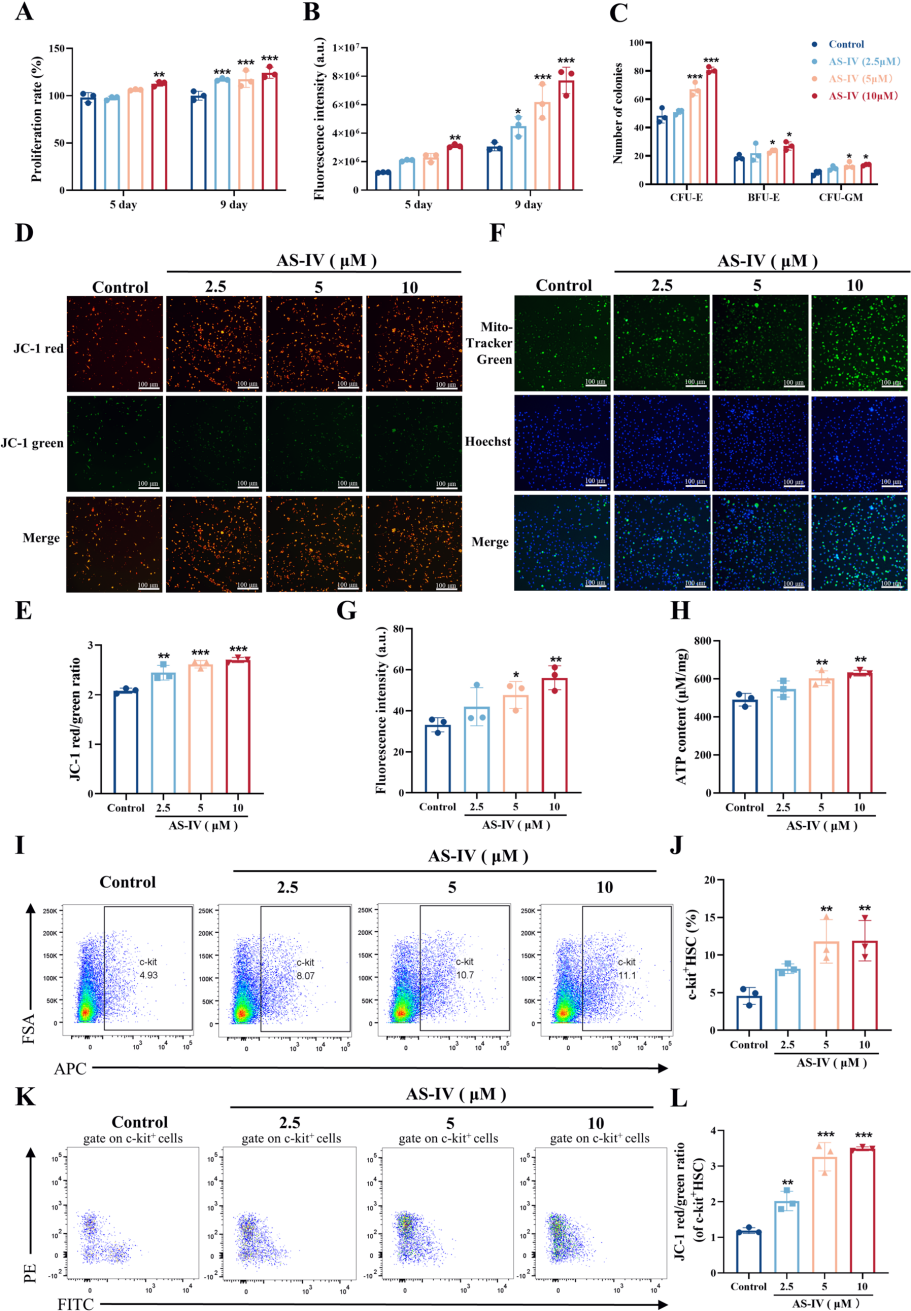
AS-IV promotes mitochondrial function and proliferation of HSC in vitro*.* **A** BMNCs were isolated from KM mice and cultured in vitro. The cell viability of bone marrow nucleated cells was assessed using the CCK-8 assay on day 5 and 9 after administration of AS-IV intervention; **B** Bone marrow nucleated cells from eGFP-fluorescent mice were extracted and cultured in vitro, and their proliferation was counted by fluorescence photography and fluorescence intensity analysis after the intervention of AS-IV administration on day 5 and 9; **C** Cell colony formation assay to detect CFU-E, BFU-E, CFU-GM proliferation after AS-IV intervention on bone marrow nucleated cells; **D**–**E** Bone marrow nucleated cells treated with AS-IV-labelled fluorescent probe JC-1 (2.5, 5, 10 μM) to assess changes in mitochondrial membrane potential by inverted fluorescence microscopy. JC-1 polymer appears as red, whereas JC-1 monomer appears as green. Scale bar: 100 μm; **F**–**G** Mito-Tracker green fluorescent probe for detection of mitochondrial mass after drug administration. Nuclei were stained with Hoechst 33,258 (scale bar: 100 μm); **H** ATP Assay Kit detected the concentration of ATP produced by cells; **I** After 9 days of in vitro culture of bone marrow nucleated cells, the expression of c-kit^+^ HSC in each group was evaluated using flow cytometry; **J** Histograms showing the percentage of c-kit^+^ HSC in each group; **K**–**L** Mitochondrial membrane potential changes of c-kit^+^ HSC detected by flow cytometry. Data represent the mean ± standard deviation of three independent experiments. *p < 0.05, **p < 0.01, ***p < 0.001 compared with control

To further investigate AS-IV's effects on HSC proliferation, we examined colony formation and mitochondrial function in c-kit^+^ HSC within BMNCs. BMNCs were isolated using density gradient centrifugation, capturing a range of cells, including mesenchymal stem cell (MSC), HSC, and endothelial progenitor cell [36]. Microscopic observations indicated that AS-IV promoted BMNCs proliferation (Supplementary Fig. 2A), which was further confirmed by the CCK-8 assay (Fig. 2A). In vitro culture of eGFP-labeled BMNCs demonstrated increased fluorescence intensity with AS-IV treatment, suggesting enhanced cell proliferation (Fig. 2B; Supplementary Fig. 2B). Colony formation assays revealed that AS-IV significantly increased CFU-E, BFU-E, and CFU-GM colonies in comparison to the control group (Fig. 2C; Supplementary Fig. 2C). Further analysis showed that AS-IV treatment led to an increase in mitochondrial membrane potential (Fig. 2D–E), an increase in mitochondrial mass (Fig. 2F–G), and improved ATP production (Fig. 2H) within BMNCs. Flow cytometry analysis indicated that AS-IV enhanced the proportion of c-kit⁺ HSC and increased the mitochondrial membrane potential of c-kit⁺ HSC (Fig. 2I–L). These results demonstrated that AS-IV enhanced mitochondrial function and stimulated the proliferation of HSC.

### AS-IV promotes hematopoietic recovery in irradiated mice

**Fig. 3:**
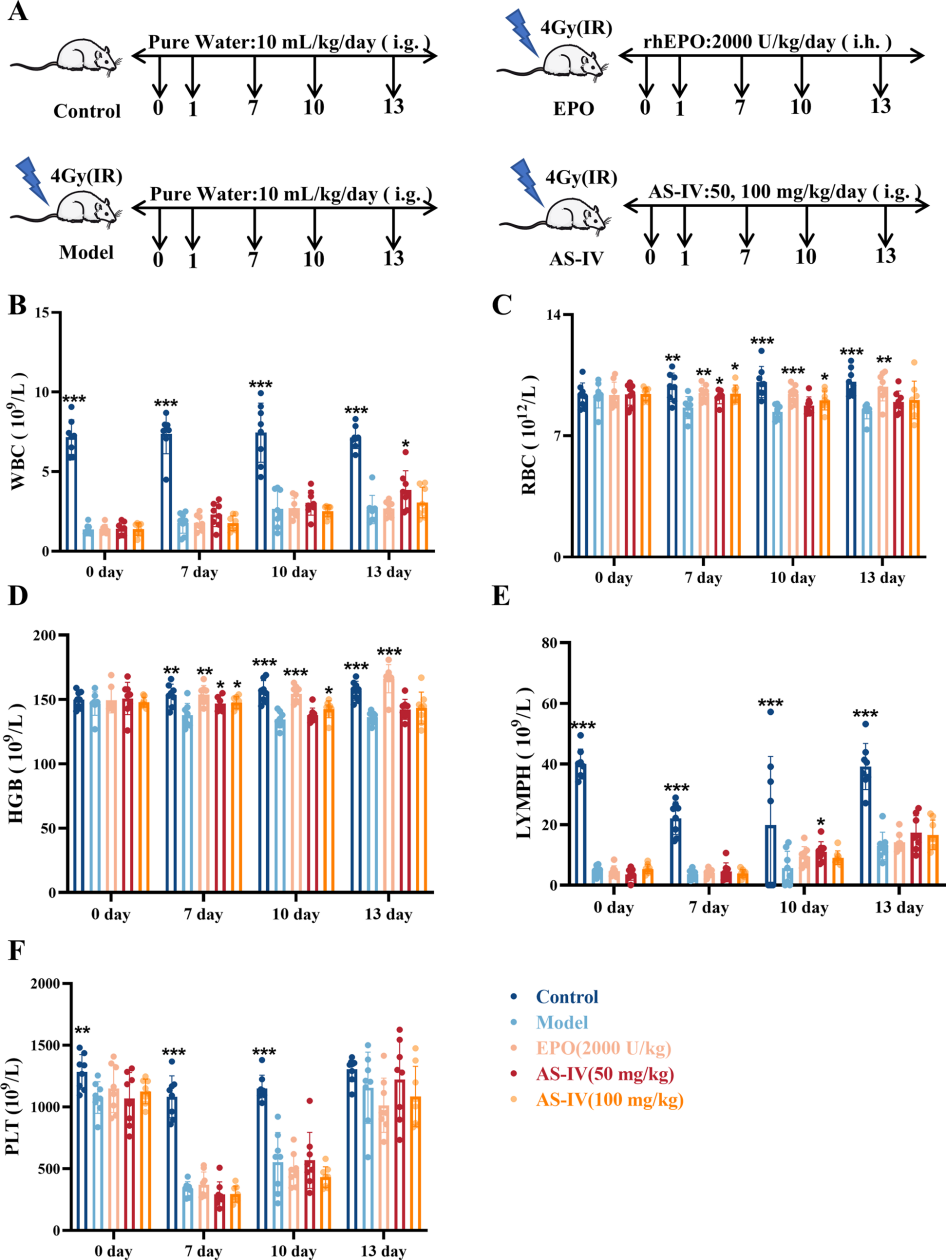
AS-IV promotes hematopoietic recovery in irradiated mice. **A** The strategies of establishing the irradiated mice and treatment of each group; **B**–**E** Blood counts showing (**B**) WBC, (**C**) RBC, (**D**) HGB, (**E**) LYMPH, and (**F**) PLT (day 0, 7, 10 and 13 after IR exposure). Data are expressed as mean ± SD (n = 8). *p < 0.05, **p < 0.01, ***p < 0.001 compared with model

Building upon the in vitro results, we conducted a further examination of the impact of AS-IV on promoting hematopoietic function recovery in a radiation-induced mouse model. Following 4.0 Gy whole-body irradiation, KM mice were administered AS-IV at doses of 50 mg/kg and 100 mg/kg every day or recombinant human EPO (2000 U/kg) every other day (Fig. 3A). The peripheral blood counts demonstrated a statistically significant reduction in white blood cells (WBC) after irradiation 24 h, confirming successful model establishment. Throughout the treatment period, leukocyte levels in the AS-IV-treated group significantly increased by day 13, with a marked rise in lymphocytes (LYMPH) observed by day 10 (Fig. 3B, 3E). The AS-IV (100 mg/kg) and EPO groups exhibited significant increases in red blood cell (RBC) counts, along with improvements in hemoglobin (HGB) levels, indicating enhanced erythropoiesis (Fig. 3C–D). The AS-IV group did not demonstrate a statistically significant impact on platelet (PLT) levels (Fig. 3F). The findings indicated that AS-IV effectively promoted the restoration of hematopoietic function, facilitating the recovery of leukocytes, lymphocytes, and erythrocytes.

### AS-IV enhances HSC mitochondrial function and promotes HSC proliferation after radiation injury

**Fig. 4:**
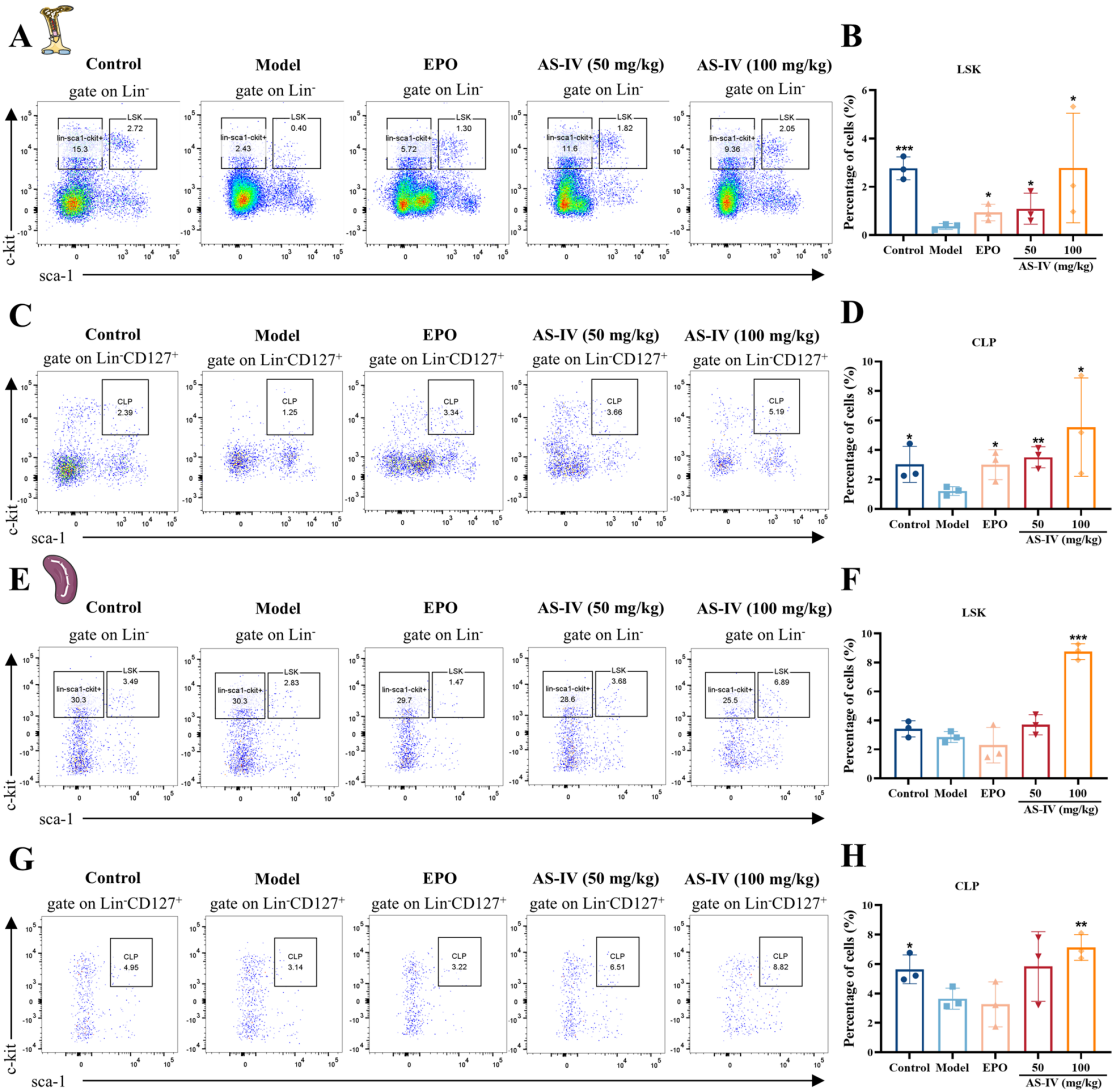
AS-IV promotes HSC proliferation after radiation injury. The expression of HSC in the BM and SP was assessed using flow cytometry 10 days post-treatment. **A**–**B** The frequencies of HSC (LSK, Lin^−^ sca-1^+^ c-kit^+^) in the BM cells of each group (n = 3); **C**–**D** The frequencies of common lymphoid progenitor cell (CLP, Lin^−^ CD127^+^ sca-1^+^ c-kit ^+^) in the BM cells of each group (n = 3); (**E**–**F**) The frequencies of HSC in the SP cells of each group (n = 3); **G**–**H** The frequencies of CLP in the SP cells of each group (n = 3). Data represent the mean ± standard deviation of three independent experiments. * p < 0.05, ** p < 0.01, *** p < 0.001 compared with model

**Fig. 5:**
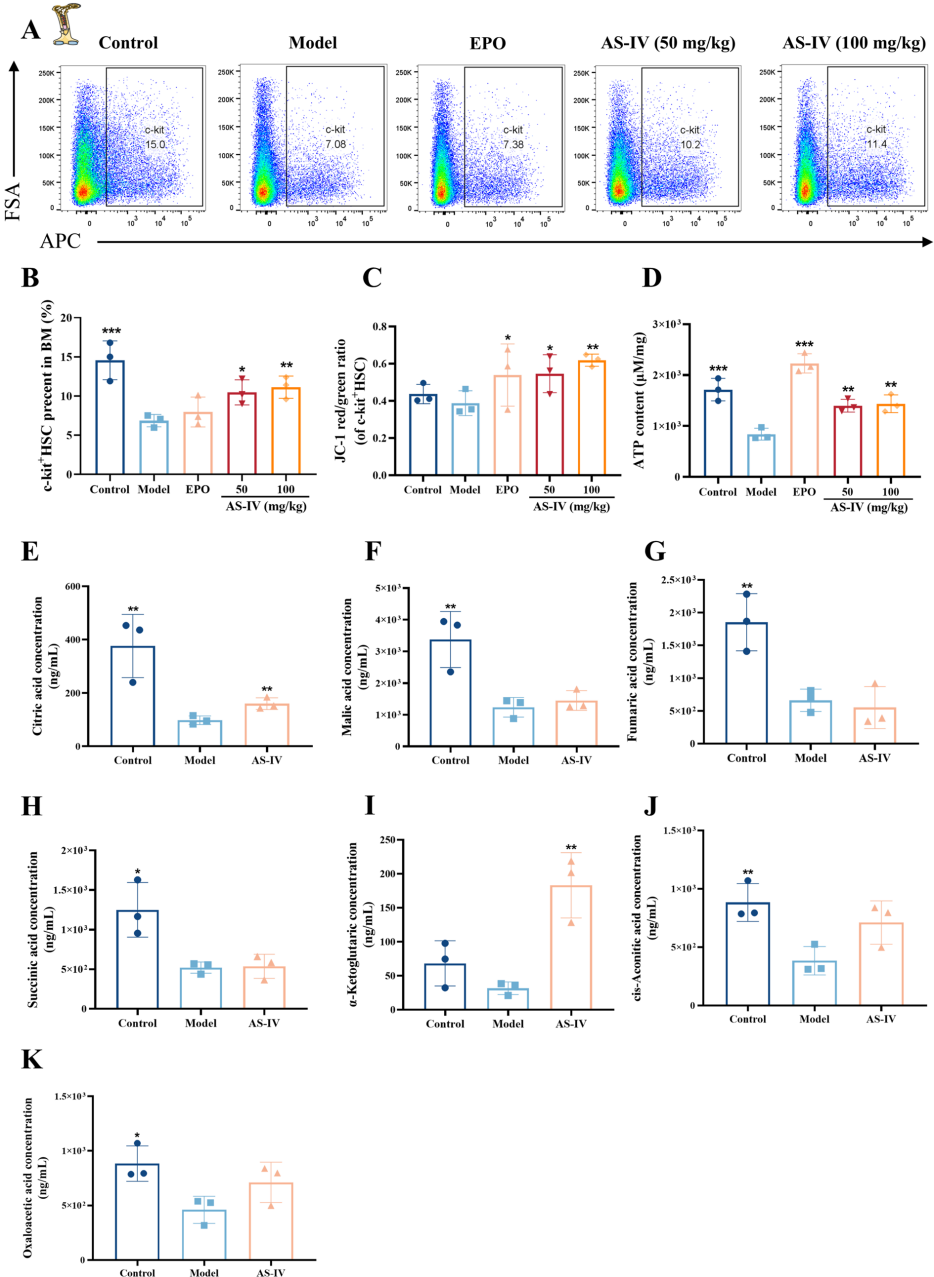
AS-IV enhances HSC mitochondrial function after radiation injury. **A** Flow cytometry was utilized to assess the proportion of c-kit^+^ HSC in the bone marrow of each group 10 days post-treatment; **B** Histograms showing the percentage of c-kit^+^ HSC in the bone marrow of each group; **C** Histograms showing the changes in mitochondrial membrane potential of c-kit^+^ HSC in the bone marrow of each group; **D** An ATP assay kit was used to detect the concentration of ATP produced by BM cells; **E**–**K** Targeted metabolomics analysis was utilized to analyze changes in the concentrations of citric acid, malic acid, fumaric acid, succinic acid, α-ketoglutaric acid, cis-aconitic acid, and oxaloacetic acid in bone marrow. Data represent the mean ± standard deviation of three independent experiments. *p < 0.05, **p < 0.01, ***p < 0.001 compared with model

Radiation-induced myelosuppression primarily damages the hematopoietic system through the destruction of hematopoietic stem and progenitor cells [37]. Flow cytometry analysis on day 10 post-irradiation showed increased proportions of LSK HSC and CLPs in the bone marrow following treatment with AS-IV and EPO (Fig. 4A–D; Supplementary Fig. 3A-D). AS-IV also enhanced the proliferation of splenic HSC (Fig. 4E–H; Supplementary Fig. 3E–H). To assess the effects of AS-IV on mitochondrial function in HSC, mitochondrial membrane potential was evaluated by flow cytometry in phenotypically characterized HSC. AS-IV (100 mg/kg) significantly increased mitochondrial membrane potential and ATP production of c-kit⁺ HSC in radiation-damaged mice compared to the model group (Fig. 5A–D; Supplementary Fig. 4A–G). Metabolomics analyses showed that the concentration of TCA cycle intermediates in bone marrow and spleen was elevated after AS-IV treatment, suggesting that AS-IV enhanced the level of mitochondrial energy metabolism in bone marrow and spleen cells (Fig. 5E–K; Supplementary Fig. 4H–O). These findings demonstrated that AS-IV improved the mitochondrial function of HSC, thereby promoting their proliferation following radiation-induced hematopoietic injury.

### AS-IV upregulates genes expression associated with hematopoietic lineage and mitochondrial function

**Fig. 6:**
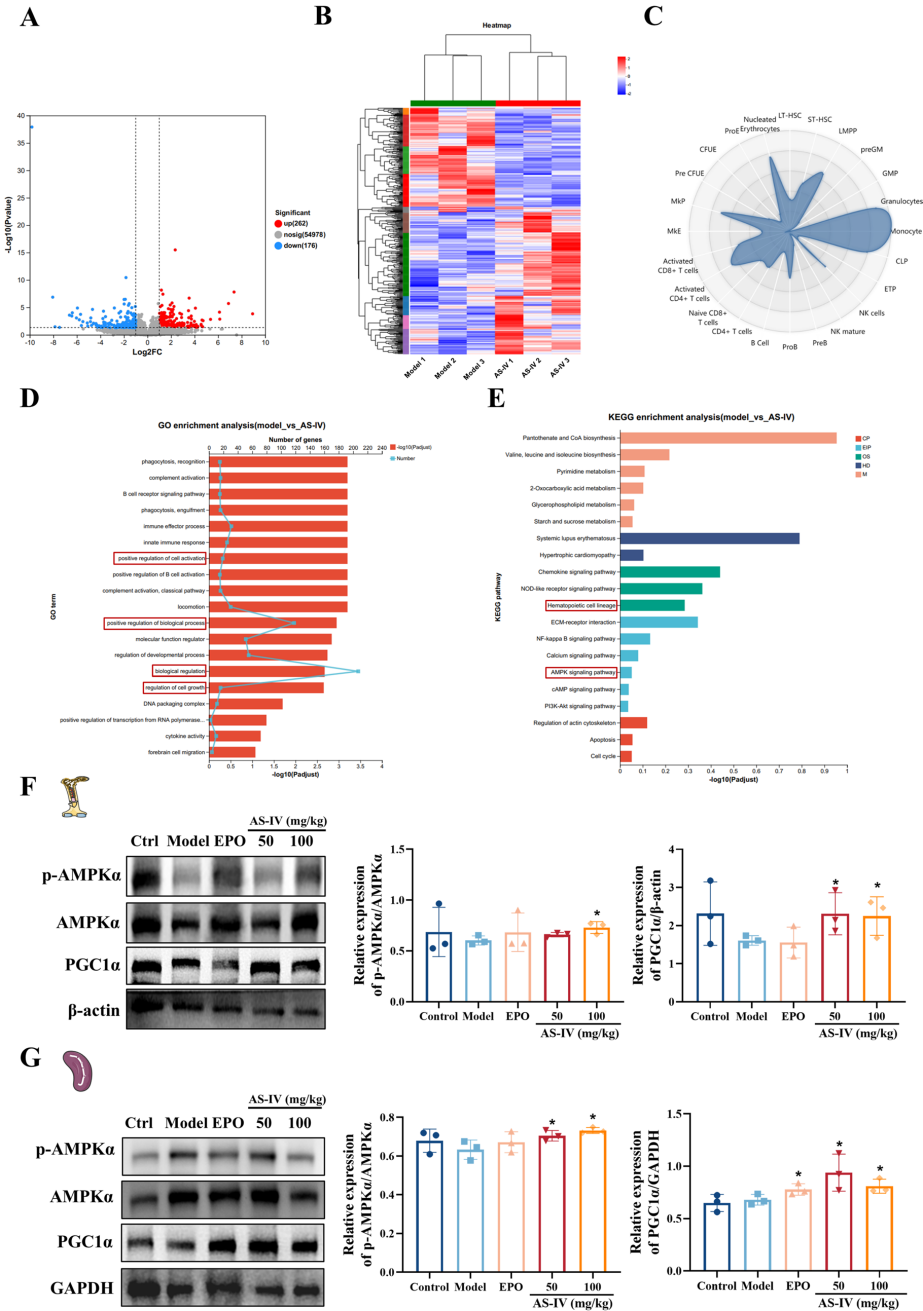
AS-IV upregulates genes express associated with hematopoietic lineage and mitochondrial function. **A** Volcano plots depicting the DEGs in both the model and AS-IV treated groups. Red dots denote upregulated genes, while blue dots indicate downregulated genes (|log_2_FC| > 1 and p-value < 0.05); **B** Hierarchical clustering analysis of AS-IV regulated DEGs; **C** Cellular radar map; **D** GO enrichment analysis of DEGs; **E** Enrichment analysis of DEGs in KEGG pathways. The positive or negative log_2_FC value, the greater the fold change in gene expression; **F**–**G** Representative immunoblot images and biochemical quantification of mitochondrial energy metabolism-related pathway proteins (AMPK/PGC1α pathway) in bone marrow and spleen of different groups of mice after irradiation injury. Data represent the mean ± standard deviation of three independent experiments. *p < 0.05, **p < 0.01, ***p < 0.001 compared with model

RNA-seq analysis of bone marrow cells derived from irradiated mice subjected to treatment with AS-IV identified 438 DEGs, comprising 262 genes that were upregulated and 176 genes that were downregulated (Fig. 6A). Clustering analysis revealed significant aggregation of DEGs in the AS-IV group, with a predominant upregulation of mRNAs (Fig. 6B). To investigate the association between gene expression changes and hematopoietic lineage cells, we utilized the CellRadar tool (https://karlssong.github.io/cellradar/), which is designed to identify cell types using publicly available lineage-related gene information. Cellular radar plots showed that AS-IV upregulated genes associated with ST-HSC, LMPP, CLP, monocytes, MKP, and nucleated erythrocyte features (Fig. 6C), indicating effects on hematopoietic stem and progenitor cells. GO enrichment analysis revealed the significant participation of DEGs in processes related to growth and development, biological regulation, and cell growth patterns, which were crucial for HSC proliferation (Fig. 6D). KEGG pathway analysis identified significant regulation by AS-IV on the hematopoietic cell lineage and AMPK signaling pathway, crucial for mitochondrial energy metabolism (Fig. 6E) [38, 39]. Then, western blot analysis confirmed the upregulation of p-AMPKα and PGC1α expression following AS-IV treatment, thereby suggesting that AS-IV enhanced mitochondrial energy metabolism through the AMPK/PGC1α pathway (Fig. 6F–G). These results indicated that AS-IV could facilitate hematopoietic cell lineage proliferation by enhancing mitochondrial function.

### AS-IV enhances HSC proliferation via the AMPK/PGC1α signaling pathway

**Fig. 7:**
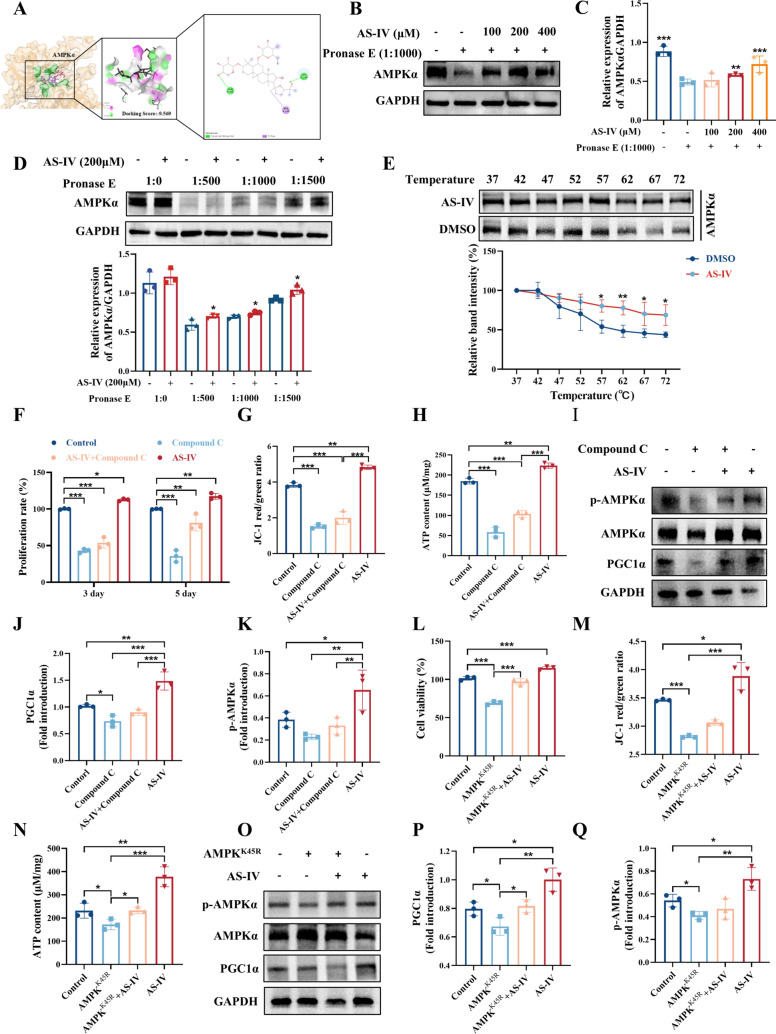
AS-IV enhances HSC proliferation via the AMPK/PGC1α signaling pathway. K562 cells were subjected to treatment with Compound C (5 μM), Compound C (5 μM) combined with AS-IV (10 μM), or AS-IV (10 μM) for a duration of 5 days. **A** Molecular docking shows the binding ability between AS-IV and its core target (AMPKα); **B**–**C** DARTS assays demonstrated dose-dependent binding of AS-IV to AMPKα in K562 cells. Treatment with Streptomyces protease (1:1000) at 40 °C for 10 min; **D** The lysates of K562 cells were treated with AS-IV (200 μM) for 1 h, followed by the addition of streptomycin (Pronase E) at various concentrations (1:500, 1:1000 or 1:1500), and incubated at 40 °C for 10 min. AMPKα content was assessed using western blot analysis; **E** CETSA analysis of AMPKα degradation damage under different temperatures. The histogram shows the relative density of AMPKα to GAPDH; **F** CCK-8 assay for K562 cell proliferation; **G** Flow cytometry analysis was conducted to assess the mitochondrial membrane potential in K562 cells; **H** ATP assay kit to detect the concentration of ATP produced by K562 cells; **I**–**K** The expression of PGC1α and p-AMPKα was detected by WB. **L** The bar graph shows the viability of K562 cells after AMPK^K45R^ gene mutation, treated with 10 μM AS-IV; **M** Flow cytometry was used to detect the changes of mitochondrial membrane potential in K562 cells with AMPK^K45R^ gene mutation. Loading or not loading AS-IV intervention; **N** ATP assay kit was used to detect the level of ATP production in K562 cells with AMPK^K45R^ mutation; **O**–**Q** Western blot was used to detect the changes of p-AMPKα and PGC1α in K562 cells with AMPK^K45R^ mutation. Data represent the mean ± standard deviation of three independent experiments. *p < 0.05, **p < 0.01, ***p < 0.001 versus the corresponding control groups

A comprehensive analysis of GO and KEGG enrichment suggested that the AMP-activated protein kinase (AMPK) pathway was a critical regulator of mitochondrial energy metabolism. Molecular docking experiments were conducted to examine the direct interaction between AS-IV and AMPKα, yielding a binding score of 9.569. This score suggested a significant affinity between AS-IV and AMPKα (Fig. 7A). To further validate this interaction, a DARTS assay was performed, revealing that AS-IV enhances the stability of AMPKα against protease-induced degradation. This finding provided additional support for the hypothesis that AS-IV interacted with AMPKα (Fig. 7B–D). Subsequently, CETSA analysis revealed that AS-IV treatment effectively stabilized AMPKα protein structure in heat-denatured K562 cell lysates across a range of thermal challenges (Fig. 7E). To elucidate the role of AS-IV in modulating mitochondrial function via the AMPK pathway, compound C was used to inhibit AMPK activity. The results indicated that compound C significantly inhibited cell proliferation, attenuated the enhancement of mitochondrial membrane potential, and decreased the elevation of ATP levels induced by AS-IV in K562 cells (Fig. 7F–H). Additionally, compound C inhibited the AS-IV-induced activation of p-AMPKα and PGC1α (Fig. 7I–K), confirming that AS-IV regulated mitochondrial function and promoted the proliferation of K562 cells through the AMPK/PGC1α signaling pathway. To interrogate AMPKα's centrality in AS-IV-mediated bioenergetic regulation, we employed dominant-negative AMPK^K45R^ mutation to inhibit AMPK phosphorylation in K562 cells (Supplementary Fig. 5A–B). AMPK^K45R^ mutation impairment profoundly attenuated AS-IV-induced cellular proliferation, increased mitochondrial membrane potential, and ATP elevation (Fig. 7L–N). Crucially, AMPK^K45R^ mutation nullified AS-IV's activation cascade, reducing p-AMPKα and PGC1α induction (Fig. 7O–Q). These findings mechanistically delineate the AMPK/PGC1α axis as the indispensable molecular transducer governing AS-IV-driven mitochondrial metabolic and proliferative potentiation in hematopoietic cells.

## Discussion

Hematopoietic cells are particularly susceptible to damage caused by irradiation or chemotherapeutic agents due to their rapid proliferation [3]. This exposure results in hematopoietic dysfunction, as evidenced by a reduction in the levels of various hematopoietic cell types. It is essential that therapeutic interventions, including chemotherapy, radiotherapy, and bone marrow transplantation, be complemented by the reestablishment of the patient's HSC proliferation and differentiation to ensure the restoration of long-term hematopoiesis [40–42]. Currently, the primary therapeutic agents for hematopoietic reconstitution following radiation or chemotherapy-induced injury are hematopoietic growth factors. These factors can quickly stimulate the proliferation of hematopoietic progenitor cells in response to short-term hematopoietic stress [43, 44]. For instance, the administration of additional EPO can facilitate the generation of erythroid progenitor cells into red blood cells. However, the prolonged use of EPO carries the risk of depleting HSC reserves [45]. Therefore, developing agents that specifically target HSC proliferation and differentiation could enhance long-term hematopoietic recovery and address the limitations of current therapies.

Mitochondrial function plays a critical role in the proliferation, differentiation, and maintenance of normal hematopoietic lineage cells within HSC [46]. HSC contain a large number of relatively inactive mitochondria, which become more active as HSC differentiate into multipotent and restricted progenitor cells. Notably, impaired mitochondrial function in HSC leads to defects in differentiation, lymphopenia, anemia, and a bias toward myeloid differentiation [47]. Recent evidence suggests that mitochondria act as signaling organelles that influence HSC fate and functionality [48, 49]. The metabolite α-ketoglutarate, a product of the mitochondrial TCA cycle, has been shown to regulate HSC differentiation through mechanisms involving histone and DNA methylation. The addition of exogenous α-ketoglutarate can promote HSC differentiation in both human and murine models. Moreover, the upregulation of OXPHOS in activated adult stem cells typically results in increased levels of α-ketoglutarate and decreased levels of succinate or L-2-hydroxyglutarate, facilitating histone and DNA demethylation and thereby promoting stem cell activation, proliferation, and differentiation [50]. Thus, modulating mitochondrial function to alleviate aging and radiation-induced hematopoietic damage in HSC represents a promising therapeutic strategy.

Efforts to enhance mitochondrial function in HSC, such as the use of coenzyme Q or niacinamide, have shown potential in counteracting the decline in HSC activity due to aging and other stressors [51, 52]. However, clinical studies have reported minimal effects of oral niacinamide riboside supplementation on mitochondrial energy metabolism. The clinical application of traditional Chinese medicine for the purposes of tonifying qi and promoting blood circulation provides empirical evidence that qi tonic drugs may contribute to the recovery of hematopoiesis by enhancing the mitochondrial function [53]. This represents a promising direction for the discovery of new therapeutic agents. AS-IV can activate AMPK signaling pathway and enhance mitochondrial function of skeletal muscle and cardiomyocytes [54, 55]. Recent studies have shown that AS-IV enhances the expression of hematopoietic factors, thereby accelerating hematopoiesis [56]. However, its direct effects on HSC have not been extensively studied.

**Fig. 8:**
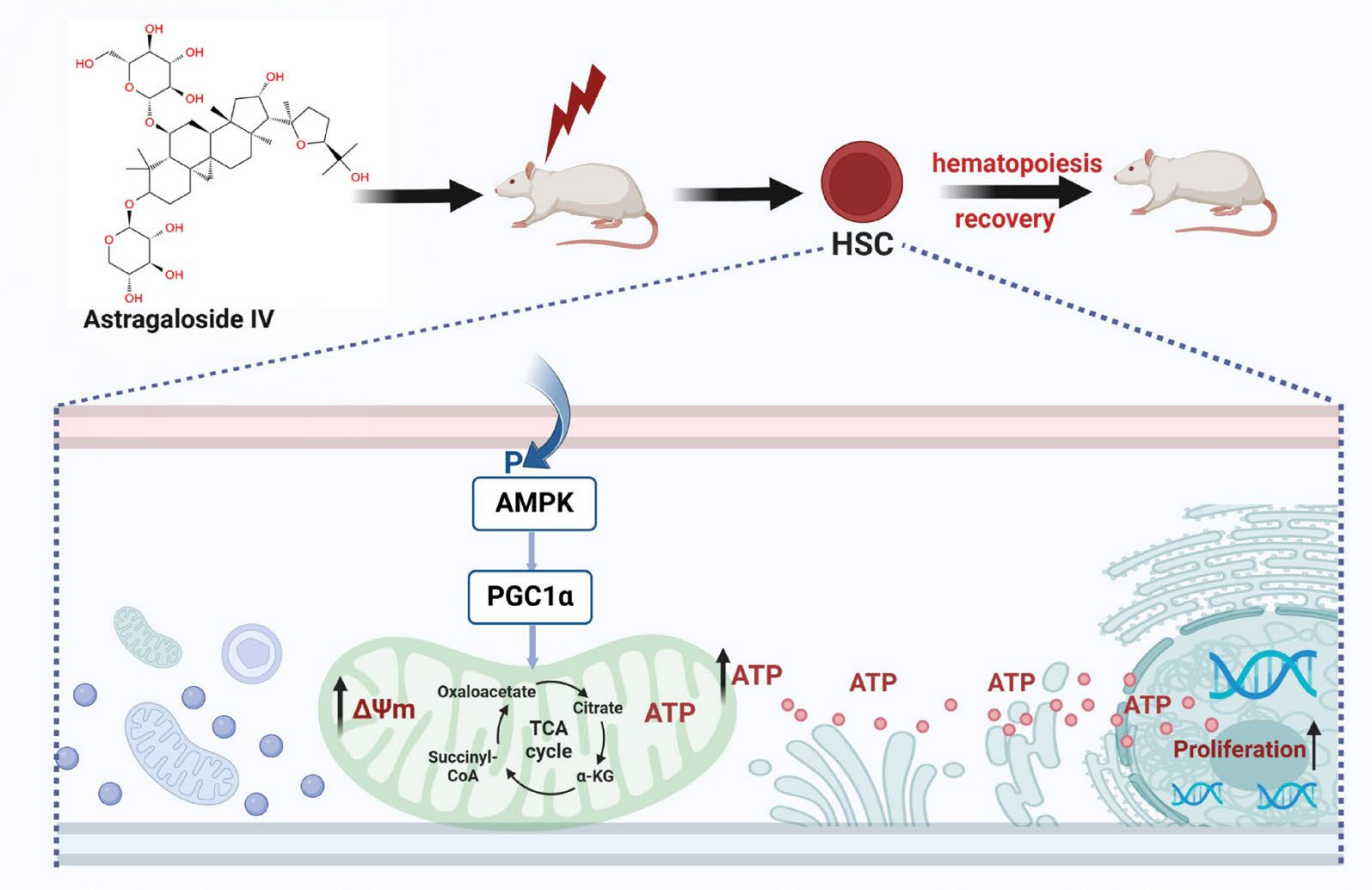
This diagram elucidates the mechanism by which AS-IV enhances the mitochondrial functionality of HSC to facilitate hematopoietic reconstruction. AS-IV activates the AMPK/PGC1α signaling pathway, thereby augmenting HSC mitochondrial function through the promotion of the TCA cycle, ATP synthesis, and an increase in mitochondrial membrane potential. The enhancement of mitochondrial function in HSC fosters their proliferation, ultimately expediting hematopoietic reconstruction

In this study, we demonstrate that AS-IV enhances mitochondrial function and augments ATP production in HSC. The AMPK signaling pathway plays a pivotal role in regulating energy metabolism and mitochondrial homeostasis, where AMPKα phosphorylates and activates PGC1α to modulate ATP production. Genomic sequencing data revealed significant enrichment in AMPK signaling pathway after AS-IV treatment. Therefore, by administering an AMPK inhibitor and employing the AMPK ^K45R^ mutation, we confirmed that AS-IV-mediated enhancement of mitochondrial function and promotion of HSC proliferation occur via the AMPK/PGC1α signaling pathway. Multiple lines of evidence demonstrate that AS-IV-mediated effects (including enhanced cardiomyocyte energy metabolism and macrophage autophagy activation) are mechanistically linked to AMPK signaling [57, 58]. Molecular docking analysis further demonstrated favorable binding affinity between AS-IV and AMPKα. Collectively, we demonstrate that AS-IV enhances mitochondrial function in HSC by activating the AMPK/PGC1α signaling pathway, thereby promoting HSC proliferation and stimulating hematopoiesis (Fig. 8).

Traditionally, *Astragalus membranaceus* has been used to boost immunity and hematopoiesis, particularly to aid in recovery following radiation therapy or chemotherapy [59]. Our study demonstrates that AS-IV, the major component of *Astragalus membranaceus*, enhances mitochondrial function in HSC through the AMPK/PGC1α pathway, thereby promoting HSC proliferation and stimulating hematopoiesis. *Astragalus membranaceus* has a long-term clinical application in China due to its efficacy and safety. AS-IV (the principal active component of *Astragalus membranaceus*) demonstrates potential as a novel mitochondrial stimulant in HSC, functioning through mechanisms that differ from those of existing hematopoietic growth factor therapies. This presents a promising therapeutic avenue for addressing functional impairments of HSC caused by chemotherapy, radiation, and aging.

## Conclusion

It is expected that improving the mitochondrial function of HSC to support hematopoietic recovery may be recognized as an innovative therapeutic approach for addressing radiation-induced hematopoietic dysfunction. AS-IV may serve as a novel mitochondrial-targeted agent that enhances mitochondrial function to promote HSC proliferation via the activation of the AMPK/PGC1α signaling pathway.

## Supplementary Information

Additional file 1

## Data Availability

The datasets are available from the corresponding author upon reasonable request.
