# Supplementary material for: Astragaloside IV accelerates hematopoietic reconstruction by improving the AMPK/PGC1α-mediated mitochondrial function in hematopoietic stem cells
Source: Chin Med. 2025 Apr 1;20:44. doi: 10.1186/s13020-025-01092-3 (PMC11963557; doi:10.1186/s13020-025-01092-3)
Supplement: Supplementary file 1 — Additional file 1 [file 13020_2025_1092_MOESM1_ESM.docx]

**Appendix A. Supplementary data**

**
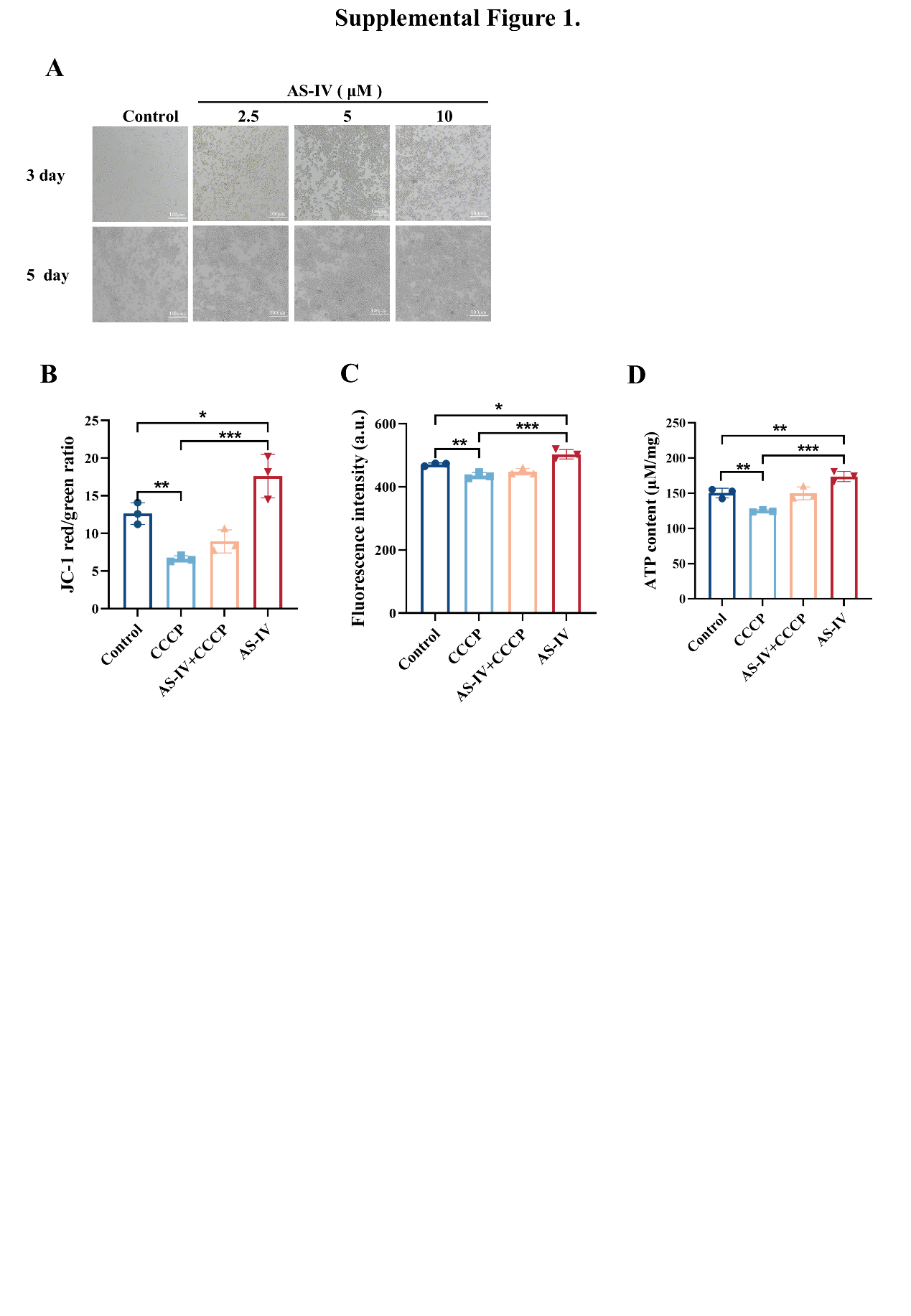
**

**Supplemental Figure 1. AS-IV enhances mitochondrial function and proliferation in K562 cells *in vitro.*** (A) Representative images of K562 cells treated with different concentrations of AS-IV (2.5, 5, and 10 μM) for 3 and 5 days. (B) Changes in mitochondrial membrane potential after loading CCCP intervention. (C) Mitochondrial mass changes after loading CCCP intervention. (D) ATP production following CCCP intervention. Data are mean ± standard deviation of three independent experiments. ** p ˂ 0.05, ** p ˂ 0.01, *** p ˂ 0.001* compared with model


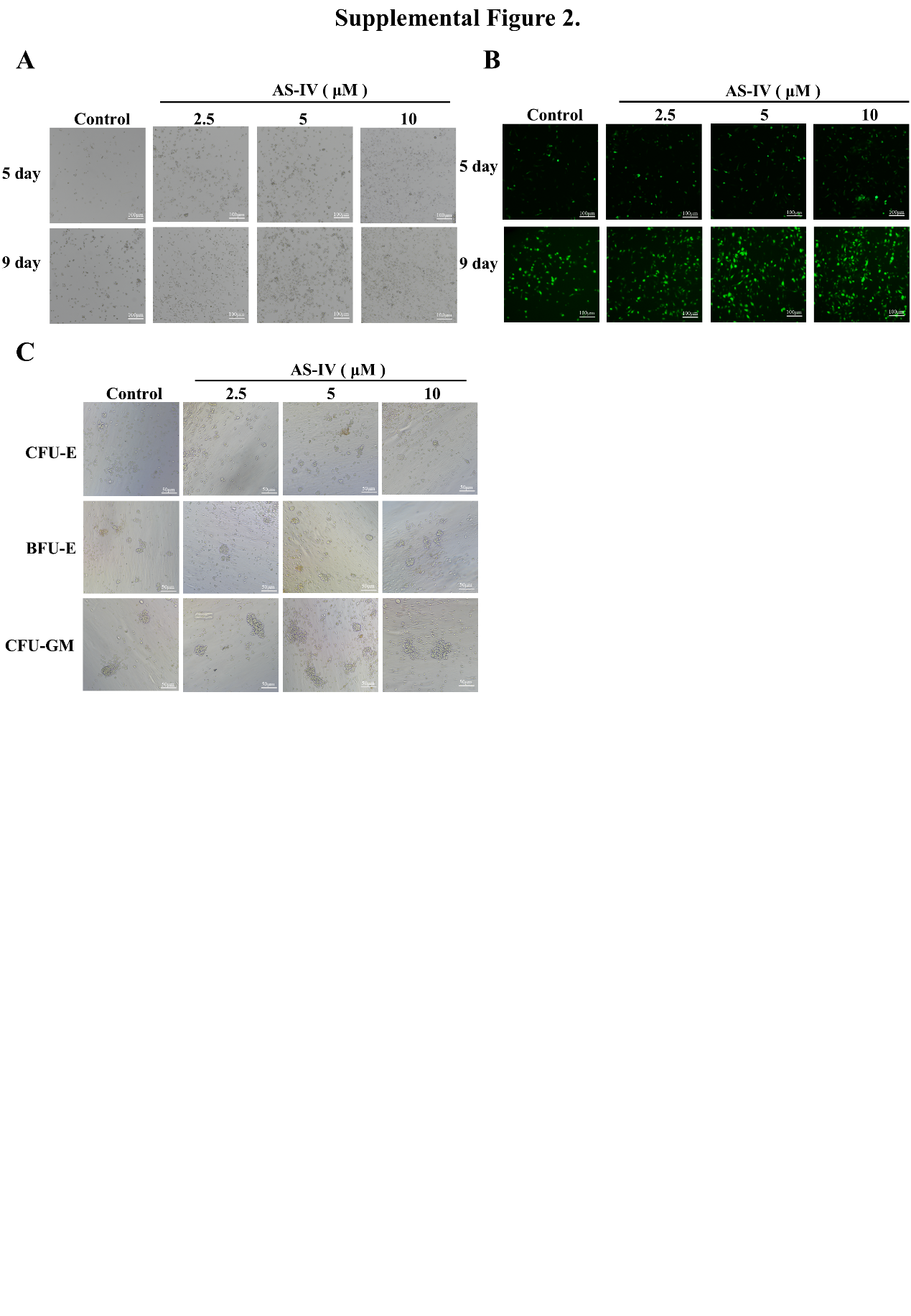


**Supplemental Figure 2.** **AS-IV promotes mitochondrial function and proliferation of HSC *in vitro.*** (A) Representative images of isolated bone marrow nucleated cells from C57BL mice treated with different concentrations of AS-IV (2.5, 5 and 10 μM) for 5 and 9 days; (B) Representative images of eGFP mouse bone marrow nucleated cells extracted in vitro and treated with different concentrations of AS-IV (2.5, 5 and 10 μM) for 5 and 9 days; (C) Cell colony formation assay to detect CFU-E (Colony-Forming Unit – Erythroid), BFU-E (Burst-Forming Unit – Erythroid), CFU-GM (Colony-Forming Unit – Granulocyte-Macrophage) proliferation after AS-IV intervention on bone marrow nucleated cells.


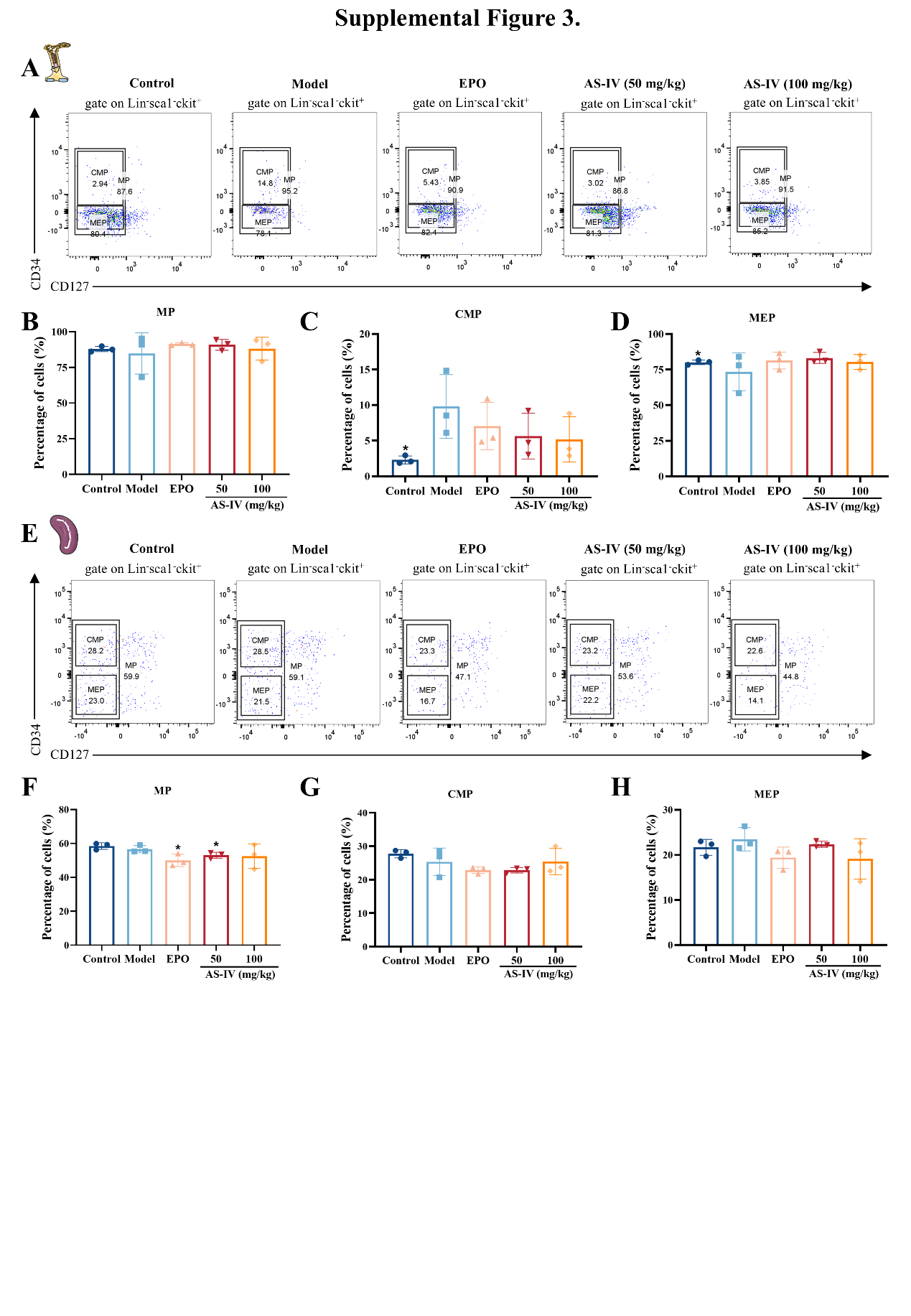


**Supplemental Figure 3. AS-IV promotes HSC proliferation** **after radiation injury.** (A-H) 10 days after treatment, flow cytometry was used to detect the expression of myeloid progenitors (MP, Lin^-^ CD127^-^ sca-1^-^ c-kit ^+^), common myeloid progenitor cell (CMP, Lin^-^ CD127^-^ sca-1^-^ c-kit ^+^ CD34^+^), and megakaryocyte-erythroid progenitor cell (MEP, Lin^-^ CD127^-^ sca-1^-^ c- kit ^+^ CD34^-^). The histogram represents the percentage of MP, CMP and MEP cells in each group. Data are mean ± standard deviation of three independent experiments. ** p < 0.05, ** p < 0.01, *** p < 0.001* compared with model


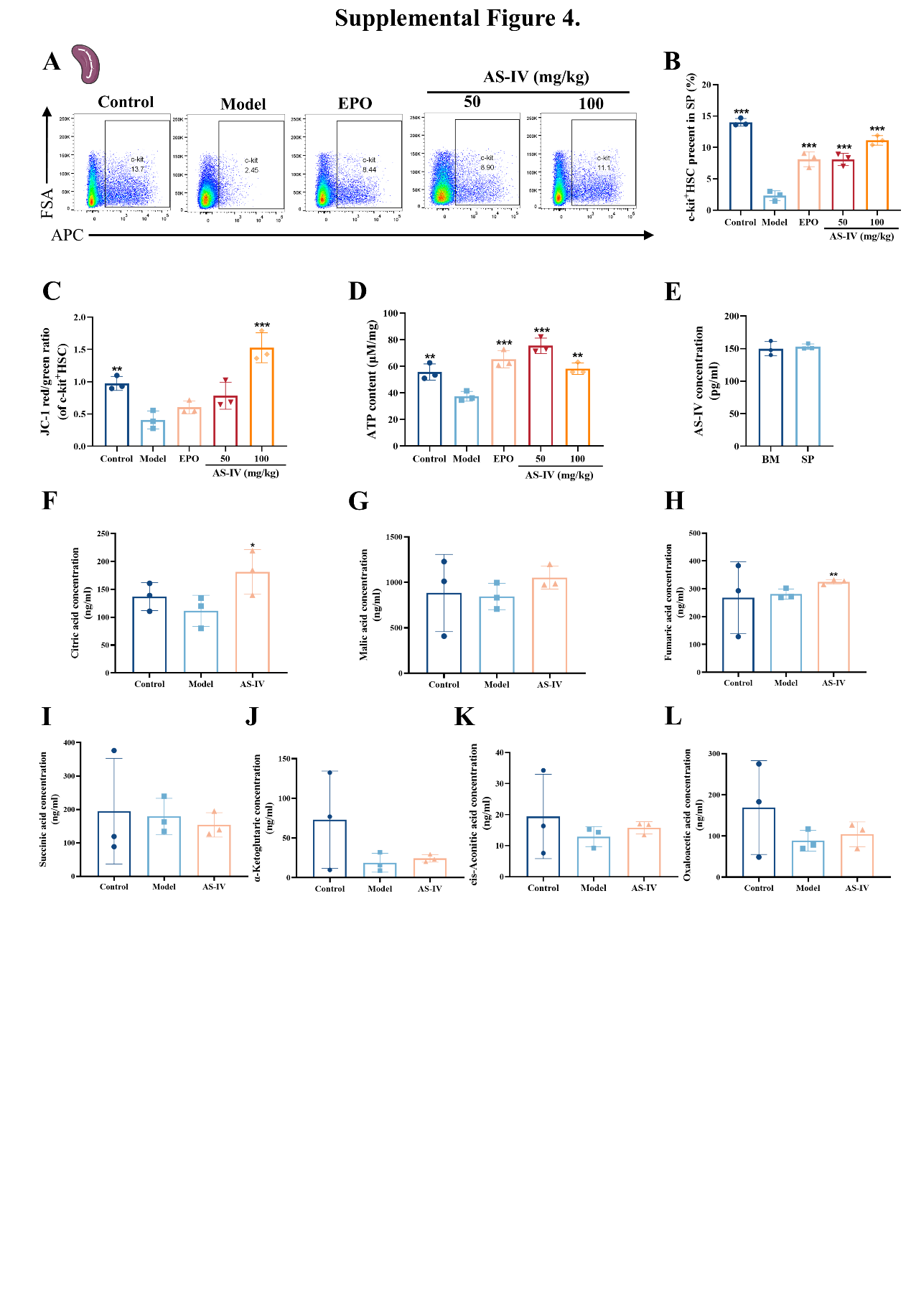


**Supplemental Figure 4. AS-IV** **enhances HSC mitochondrial function after radiation injury.** (A) Expression of c-kit ^+^ HSC in the spleen of each group was detected by flow cytometry 10 days after treatment ; (B) Histograms showing the percentage of c-kit ^+^HSC in the spleen of each group; (C) Histograms showing the changes in mitochondrial membrane potential of c-kit ^+^HSC in the spleens of each group; (D) Changes in ATP content in the splenic marrow as determined by the ATP kit; (E) Targeted metabolomics analysis quantified AS-IV in bone marrow and spleen; (F-L) Targeted metabolomics analysis to determine changes in the levels of citric acid, malic acid, fumaric acid, succinic acid, α-ketoglutaric acid, cis-aconitic acid and oxaloacetic acid in the spleen; Data are mean ± standard deviation of three independent experiments. ** p < 0.05, ** p < 0.01, *** p < 0.001* compared with model

*
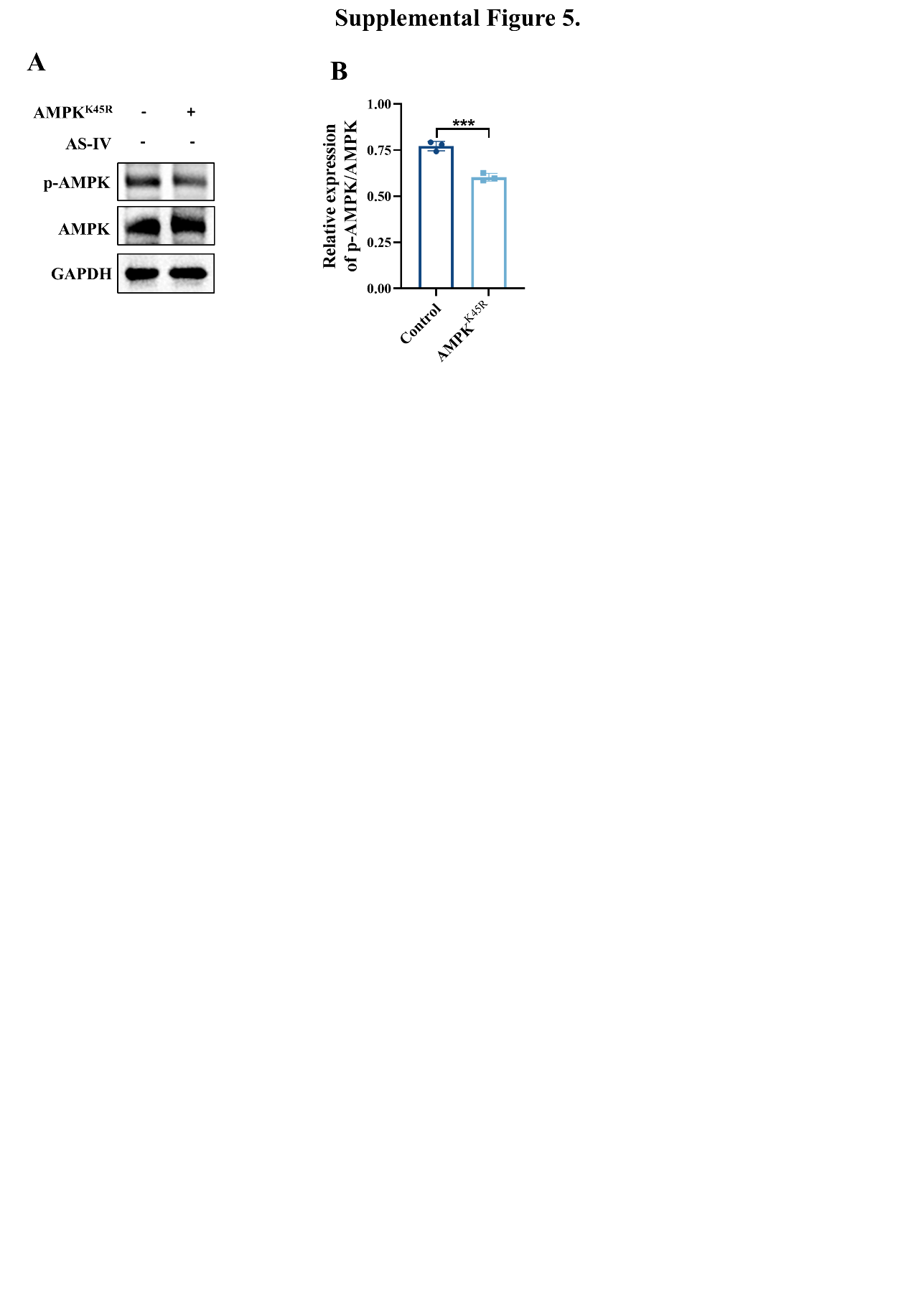
*

**Supplemental Figure 5. AS-IV enhances HSC proliferation via the AMPK/PGC1α signaling pathway.** (A-B). Western blot was used to verify the changes of p-AMPK in K562 cells with AMPK^K45R^ mutation. Data represent the mean ± standard deviation of three independent experiments. ** p < 0.05, ** p < 0.01, *** p < 0.001* compared with model

**Supplemental Table 1. Primer sequences for qRT-PCR.**

| **Gene name** | **Primer sequence（5'→3'）** |
| --- | --- |
| TFAM Forward | TTTCTCCGAAGCATGTGGGG |
| TFAM Reverse | CTTCAGCTTTTCCTGCGGTG |
| NRF1 Forward | GGGCGGGAAGACCTTTTGTA |
| NRF1 Reverse | TCAGTCAGGATCCACTTGCG |
| SDH Forward | CTGGAGATCCGAGAAGGAAGAG |
| SDH Reverse | AGCGAAGATCATGGCTGTCTC |
| COXII Forward | CTATCCTGCCCGCCATCATC |
| COXII Reverse | GATTAGTCCGCCGTAGTCGG |
| SOD2 Forward | TGAACAATCTCAACGCCACCGAG |
| SOD2 Reverse | TGAACTTCAGTGCAGGCTGAAGAG |
| GAPDH Forward | TGTGGGCATCAATGGATTTGG |
| GAPDH Reverse | ACACCATGTATTCCGGGTCAAT |
